# Supplementary material for: The AP-2 complex interacts with γ-TuRC and regulates the proliferative capacity of neural progenitors
Source: Life Sci Alliance. 2023 Dec 12;7(2):e202302029. doi: 10.26508/lsa.202302029 (PMC10716017; doi:10.26508/lsa.202302029)
Supplement: Supplementary file 3 [file LSA-2023-02029_SdataFS2.pptx]

## Slide 1
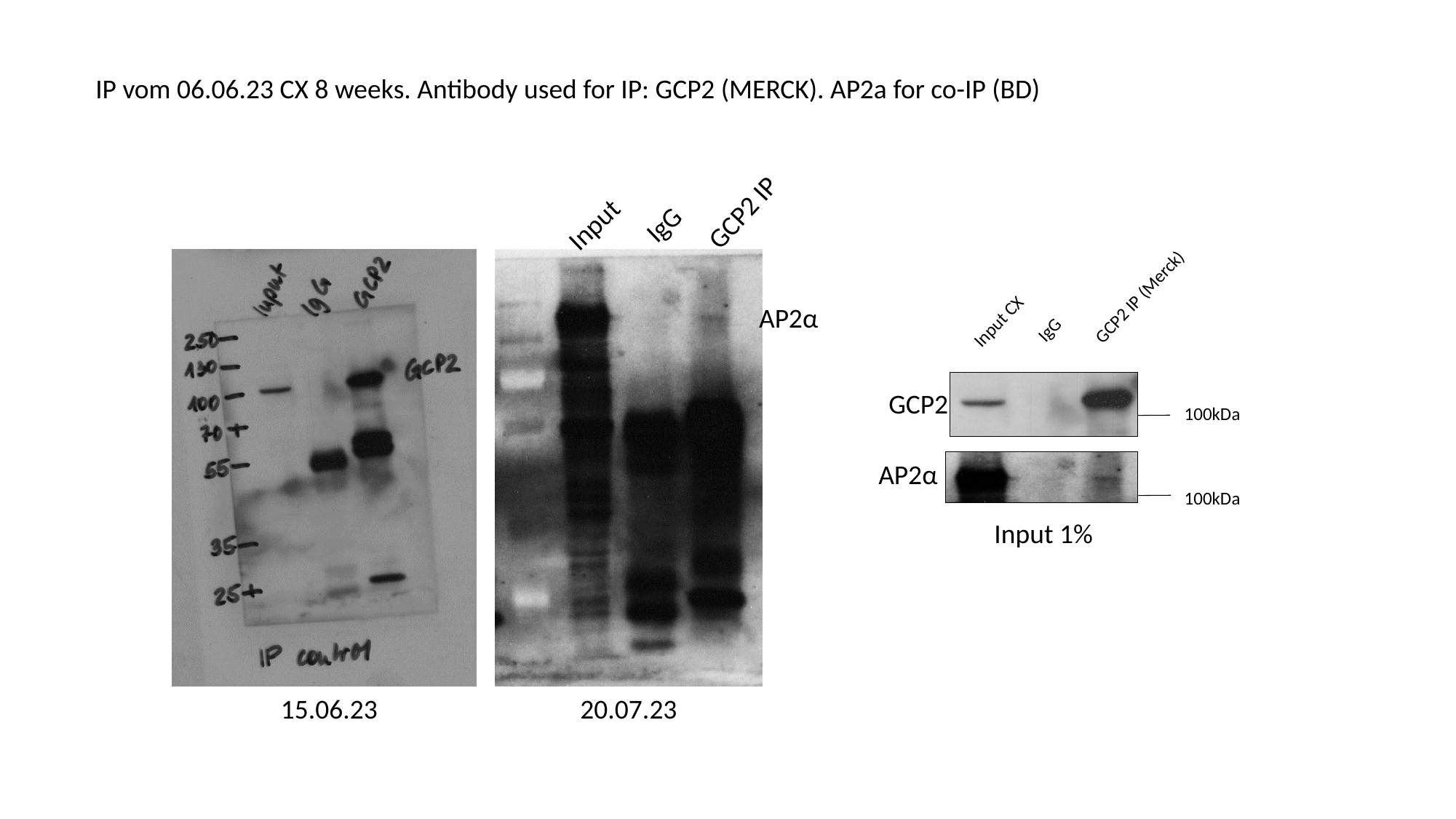

IP vom 06.06.23 CX 8 weeks. Antibody used for IP: GCP2 (MERCK). AP2a for co-IP (BD)
GCP2 IP
Input
IgG
GCP2 IP (Merck)
AP2α
Input CX
IgG
GCP2
100kDa
AP2α
100kDa
Input 1%
15.06.23
20.07.23
