# Supplementary material for: The AP-2 complex interacts with γ-TuRC and regulates the proliferative capacity of neural progenitors
Source: Life Sci Alliance. 2023 Dec 12;7(2):e202302029. doi: 10.26508/lsa.202302029 (PMC10716017; doi:10.26508/lsa.202302029)
Supplement: Supplementary file 6 [file LSA-2023-02029_Supplemental_Data_1.docx]

Appendix

**The AP-2 complex interacts with γ-TuRC and regulates the proliferative capacity of neural progenitors**

Santiago Camblor-Perujo^1§^, Ebru Ozer Yildiz^1§^, Hanna Küpper^1^, Melina Overhoff ^1,2^, Saumya Rastogi^1^, Hisham Bazzi^1,3,4^, Natalia L. Kononenko^1,2,3,5*^

^1^ CECAD Excellence Center, University of Cologne, Germany, D-50931.

^2^ Center for Physiology, Faculty of Medicine and University Hospital Cologne, University of Cologne, Germany, D-50931

^3^ Center for Molecular Medicine Cologne, Faculty of Medicine and University Hospital Cologne, University of Cologne, Germany, D-50931

^4^Department of Dermatology and Venereology, Faculty of Medicine and University Hospital Cologne, University of Cologne, Germany, D-50931

^5^Institute of Genetics, Natural Faculty, University of Cologne, Germany, D-50931.

§ Equal contribution

*Corresponding author: n.kononenko@uni-koeln.de

**Appendix Table S1:** Primers used for genotyping of genetically modified mice used in the current study.

| Gene | Sequence (5'- 3') |
| --- | --- |
| *Ap2m1 1* | CTC ATA TAC GAG CTG CTG GAT G |
| *Ap2m1 2* | CCA AGG GAC CTA CAG GAC TTC |
| *Cre 1* | GAA CCT GAT GGA CAT GTT CAG G |
| *Cre 2* | AGT GCG TTC GAA CGC TAG AGC CTG T |
| *Cre 3* | TTA CGT CCA TCG TGG ACA |
| *Cre 4* | TGG GCT GGG TGT TAG CC |
