# Supplementary material for: The AP-2 complex interacts with γ-TuRC and regulates the proliferative capacity of neural progenitors
Source: Life Sci Alliance. 2023 Dec 12;7(2):e202302029. doi: 10.26508/lsa.202302029 (PMC10716017; doi:10.26508/lsa.202302029)
Supplement: Supplementary file 7 [file LSA-2023-02029_Supplemental_Data_2.docx]

Appendix

**The AP-2 complex interacts with γ-TuRC and regulates the proliferative capacity of neural progenitors**

Santiago Camblor-Perujo^1§^, Ebru Ozer Yildiz^1§^, Hanna Küpper^1^, Melina Overhoff ^1,2^, Saumya Rastogi^1^, Hisham Bazzi^1,3,4^, Natalia L. Kononenko^1,2,3,5*^

^1^ CECAD Excellence Center, University of Cologne, Germany, D-50931.

^2^ Center for Physiology, Faculty of Medicine and University Hospital Cologne, University of Cologne, Germany, D-50931

^3^ Center for Molecular Medicine Cologne, Faculty of Medicine and University Hospital Cologne, University of Cologne, Germany, D-50931

^4^Department of Dermatology and Venereology, Faculty of Medicine and University Hospital Cologne, University of Cologne, Germany, D-50931

^5^Institute of Genetics, Natural Faculty, University of Cologne, Germany, D-50931.

§ Equal contribution

*Corresponding author: n.kononenko@uni-koeln.de

**Appendix Table S2:** Plasmid DNA used for transfection of cells *in-vitro* in the current study.

| Plasmid (source gene) | Manufacturer | Identifier |
| --- | --- | --- |
| pmCherry-N1 | Clontech | 632524 |
| EB3-tdTomato (human) | Addgene | #50708 |
| AP-2µ-mCherry | Addgene | #27672 |
| γ-Tubulin-S65T-GFP | Kind gift from Dr. H. Bazzi | N/A |
| AP-2-IRES-mRFP | custom-made | Kononenko et al., 2017 |
| scramble shRNA | custom-made | Kononenko et al., 2014 |
| CHC shRNA | custom-made | Kononenko et al., 2014 |
