# Supplementary material for: The AP-2 complex interacts with γ-TuRC and regulates the proliferative capacity of neural progenitors
Source: Life Sci Alliance. 2023 Dec 12;7(2):e202302029. doi: 10.26508/lsa.202302029 (PMC10716017; doi:10.26508/lsa.202302029)
Supplement: Supplementary file 8 [file LSA-2023-02029_Supplemental_Data_3.docx]

Appendix

**The AP-2 complex interacts with γ-TuRC and regulates the proliferative capacity of neural progenitors**

Santiago Camblor-Perujo^1§^, Ebru Ozer Yildiz^1§^, Hanna Küpper^1^, Melina Overhoff ^1,2^, Saumya Rastogi^1^, Hisham Bazzi^1,3,4^, Natalia L. Kononenko^1,2,3,5*^

^1^ CECAD Excellence Center, University of Cologne, Germany, D-50931.

^2^ Center for Physiology, Faculty of Medicine and University Hospital Cologne, University of Cologne, Germany, D-50931

^3^ Center for Molecular Medicine Cologne, Faculty of Medicine and University Hospital Cologne, University of Cologne, Germany, D-50931

^4^Department of Dermatology and Venereology, Faculty of Medicine and University Hospital Cologne, University of Cologne, Germany, D-50931

^5^Institute of Genetics, Natural Faculty, University of Cologne, Germany, D-50931.

§ Equal contribution

*Corresponding author: n.kononenko@uni-koeln.de

Appendix Table S3: Antibodies & DNA labelling dyes and their used concentration in Immunoblotting (WB) and Immunocytochemistry (ICC) analysis.

| Antibody | Host | WB | ICC | Manufacturer | Identifier |
| --- | --- | --- | --- | --- | --- |
| anti-AP-2α | Mouse monoclonal | - | 1:500 | Abcam | ab2730 |
| anti-AP-2α | Mouse monoclonal | 1:1000 | - | BD | 610501 |
| anti-AP2-µ | Mouse monoclonal | - | 1:300 | BD | 611350 |
| Anti-Dcx | Guinea pig polyclonal | - | 1:500 | Merck | AB2253 |
| anti-GAPDH | Mouse monoclonal | 1:1000 | - | Sigma-Aldrich | G8795 |
| anti-GCP2 | Rabbit polyclonal | 1:1000 | 1:500 | Millipore | MABT1322 |
| anti-GCP2 | Mouse monoclonal | 1:1000 | - | Novus Biologicals | NBP2-21793 |
| anti-GCP3 | Rabbit polyclonal | 1:1000 | 1:500 | Elabscience (Biomol GmbH) | E-AB-62346 |
| anti-GCP4 | Mouse monoclonal | 1:100 | 1:50 | Santa Cruz Biotechnology | sc-271876 |
| anti-CEP135 | Rabbit polyclonal | - | 1:750 | Abcam | Ab75005 |
| anti-GFP | Chicken polyclonal | - | 1:2000 | Abcam | ab13970 |
| anti-mCherry | Mouse monoclonal | - | 1:200 | Novus Biologicals | NBP1-96752 |
| anti-Nestin | Chicken polyclonal | - | 1:500 | Novus | NB100-16074 |
| anti-p53 | Rabbit polyclonal | - | 1:1000 | CST | 2524 |
| anti-PCM1 | Rabbit polyclonal | - | 1:100 | Sigma | HPA023370 |
| anti-PCNT | Rabbit polyclonal | - | 1:500 | Abcam | ab4448 |
| anti-pHH3 | Rabbit polyclonal | - | 1:1000 | Millipore | 06-570 |
| anti-Vimentin | Chicken polyclonal | - | 1:500 | Novus Biochemical | NB300-223SS |
| anti-α-TUBULIN | Mouse monoclonal | - | 1:500 | Synaptic Systems | 302 211 |
| anti-β-actin | Mouse monoclonal | 1:1000 | - | Sigma | A-5441 |
| anti-γ-H2AX | Rabbit polyclonal | - | 1:500 | Cell Signaling | 9718 |
| anti-γ-Tubulin | Mouse monoclonal | 1:1000 | 1:1000 | Sigma | T6557 |
| Normal Mouse IgG | Normal Mouse IgG | 1:5000 | - | Millipore | 12-371 |
| Normal Rabbit IgG | Normal Rabbit IgG | 1:5000 | - | Cell Signaling | 2729 |
| Goat anti-Mouse IgG (H+L) peroxidase-conjugated | Goat anti-Mouse IgG (H+L) peroxidase-conjugated | 1:2000 | - | Jackson ImmunoResearch | 115-035-003 |
| Goat anti-Mouse IgG, light chain specific, peroxidase-conjugated | Goat anti-Mouse IgG, light chain specific, peroxidase-conjugated | 1:2000 | - | Jackson ImmunoResearch | 115-035-174 |
| Goat anti-Rabbit IgG (H+L) peroxidase-conjugated | Goat anti-Rabbit IgG (H+L) peroxidase-conjugated | 1:2000 | - | Jackson ImmunoResearch | 111-035-003 |
| Alexa Fluor 488 Goat anti-Chicken IgG | Alexa Fluor 488 Goat anti-Chicken IgG | - | 1:500 | Thermo Fisher Sci | A-11039 |
| Alexa Fluor 488 Goat anti-Mouse IgG | Alexa Fluor 488 Goat anti-Mouse IgG | - | 1:500 | Thermo Fisher Sci | A-11029 |
| Alexa Fluor 488 Goat anti-Rabbit IgG | Alexa Fluor 488 Goat anti-Rabbit IgG | - | 1:500 | Thermo Fisher Sci | A-11034 |
| Alexa Fluor 568 Goat anti-Mouse IgG | Alexa Fluor 568 Goat anti-Mouse IgG | - | 1:500 | Thermo Fisher Sci | A-11031 |
| Alexa Fluor 568 Goat anti-Rabbit IgG | Alexa Fluor 568 Goat anti-Rabbit IgG | - | 1:500 | Thermo Fisher Sci | A-11036 |
| Alexa Fluor 568 Goat anti-Chicken IgG | Alexa Fluor 568 Goat anti-Chicken IgG | - | 1:500 | Thermo Fisher Sci | A-11041 |
| Alexa Fluor 647 Goat anti-Mouse IgG | Alexa Fluor 647 Goat anti-Mouse IgG | - | 1:500 | Thermo Fisher Sci | A-21236 |
| Alexa Fluor 647 Goat anti-Rabbit IgG | Alexa Fluor 647 Goat anti-Rabbit IgG | - | 1:500 | Thermo Fisher Sci | A-21245 |
